# A new unique leafhopper genus of Erythroneurini from Thailand, with the description of one new species (Hemiptera, Cicadellidae, Typhlocybinae)

**DOI:** 10.3897/zookeys.829.28718

**Published:** 2019-03-11

**Authors:** Yuehua Song, Can Li

**Affiliations:** 1 School of Karst Science, Guizhou Normal University/ State Engineering Technology Institute for Karst Desertification Control, Guiyang, Guizhou 550001, China Guizhou Normal University Guiyang China; 2 Guizhou Provincial Key Laboratory for Rare Animal and Economic Insect of the Mountainous Region, Guiyang University, Guiyang, Guizhou 550005, China Guiyang University Guiyang China

**Keywords:** Auchenorrhyncha, Homoptera, morphology, new taxa, taxonomy

## Abstract

A new genus of the leafhopper tribe Erythroneurini (Cicadellidae, Typhlocybinae) from Thailand, Undulivena**gen. n.**, and a new species *Undulivenathaiensis***sp. n.**, are described and illustrated. The new genus exhibits a unique feature of the forewing venation with CuA vein strongly sinuate.

## Introduction

ErythroneuriniYoung (1952) is the largest tribe in the subfamily Typhlocybinae. The tribe is particularly diverse in Southeast Asia where many genera and species remain to be described. In this study, a new genus from Thailand, similar to *Salka* Dworakowska, 1972, is described based on its unique strongly sinuate CuA vein in the forewing

## Materials and methods

Morphological terminology used in this work follows Dietrich (2005). Habitus photographs were taken using a Canon EOS 5D Mark II camera and the Camlift V2.7.0 software. Multiple photographs of each specimen were compressed into final images with Zerene Stacker (64-bit) software. Body length was measured from the apex of vertex to the tip of forewings. Abdomens were removed from specimens and cleared in cold 10% KOH solution overnight. The cleared material was rinsed with water and stored in glycerin. An Olympus SZX12 dissecting microscope was used for specimen study and Olympus BX41 and BX53 stereoscopic microscopes were used alternately for drawing of the dissected male genitalia and wings. The holotype of the new species is deposited at the Queen Sirikit Botanical Garden (**QSBG**), Chiang Mai, Thailand, and additional specimens examined are deposited at the Illinois Natural History Survey (**INHS**), Prairie Research Institute, University of Illinois at Urbana-Champaign, USA (**UIUC**), and the School of Karst Science (**SKS**), Guizhou Normal University, Guiyang, China.

## Taxonomy

### Undulivena gen. n.

Taxon classificationAnimaliaHemipteraCicadellidae

http://zoobank.org/A6F8BDA7-F19C-4529-808F-AB51C1C10B16

#### Type species.

*Undulivenathaiensis* sp. n.

#### Diagnosis.

The new genus is quite different from the other genera of the tribe Erythroneurini in view of the forewing venation, patterns of patches and chaetotaxy of the subgenital plate. The CuA vein of forewing is waved, which is unique among known Erythroneurini.

#### Description.

Body yellow to beige with dark brown markings.

Head in dorsal view roundly produced, somewhat narrower than pronotum. Vertex usually with large median apical spot; coronal suture present or indistinct. Face with frontoclypeus and anteclypeus relatively slender. Pronotum broad, with posterior margin concave. Scutellum almost entirely dark, with obvious transverse impression. Forewing with claval vein distinct; MP vein slightly curved, confluent with R vein basally; CuA vein strongly sinuate. Hind wing with RA vein present.

Male abdominal apodemes small, not exceeding 3^rd^ sternite.

Male genitalia. Male pygofer lobe with posterior margin rounded, with one dorsal macrochaeta, several basolateral spine-like setae in distinct group and some similar scattered setae slightly more dorsally; dorsal appendage movably articulated basally, not extended beyond pygofer apex, connected with an extension of anal tube appendage articulated basally. Extension of anal tube appendage connected subbasally by ligament to sharp distal corners of aedeagal dorsal apodeme. Subgenital plate with few macrosetae laterally in basal half and numerous short stout setae on or near lateral and apical margin in lateral view. Style with 2^nd^ extension long, with few basal teeth on outer margin; preapical lobe distinct. Connective Y-shaped, with central lobe well developed. Aedeagal shaft very short, strongly laterally compressed, gonopore sub-apical on ventral surface; basal apodeme very short; preatrium well developed.

#### Distribution.

Thailand.

#### Remarks.

The new genus is very similar to *Salka* (from Oriental and Palearctic regions) in body shape and male genitalia, e.g., pygofer with dorsal appendages, long dorsal macrosetae and a group of basolateral macrosetae, and the presence of a median anterior lobe on the connective. It differs from *Salka* in having the venation of the forewing with CuA strongly sinuate, which is unique among known Typhlocybinae, and the subgenital plate with a few lateral macrosetae in basal half. The color pattern of the forewing is also very unusual with veins margined with yellowish white, contrasting with the dark wings.

#### Etymology.

The new generic name combines the Latin words undula and vena, referring to the undulate vein for the sinuate CuA vein of the forewing. The gender is feminine.

### Undulivena thaiensis sp. n.

Taxon classificationAnimaliaHemipteraCicadellidae

http://zoobank.org/A27A32A4-3FB2-40E6-86A5-C72B638AE804

Figs 1 –22

#### Diagnosis.

The forewing has yellow-whitish stripes along veins. The style apex expanded, with inner margin tooth-like medially, and the aedeagal shaft spindle-shaped in ventral view, with single small subbasal process.

#### Description.

**Figures 1–11.**
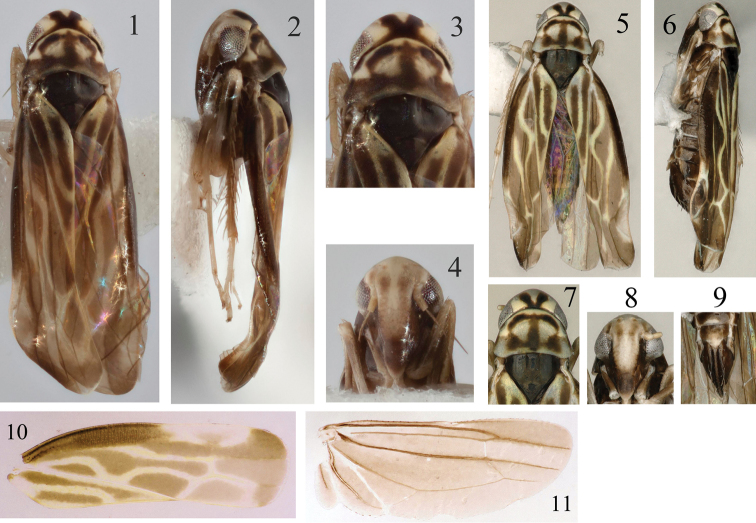
*Undulivenathaiensis* sp. n. (♂): **1** habitus, dorsal view **2** habitus, lateral view **3** head and thorax, dorsal view **4** face. (♀): **5** habitus, dorsal view **6** habitus, lateral view **7** head and thorax, dorsal view **8** face **9** abdomen of female **10** forewing **11** hind wing.

Crown yellow, with large irregular central blackish brown patch (Figs 3, 7). Eyes grey (Figs 2, 6). Face pale beige marked with brown on postclypeus laterally and on anteclypeus basally (Figs 4, 8). Pronotum whitish yellow with large patch medially and hind margin, blackish brown (Fig. 1). Scutellum blackish brown (Fig. 1). Fore wing dark brown with veins margined with yellow-white (Fig. 1).

**Figures 12–23.**
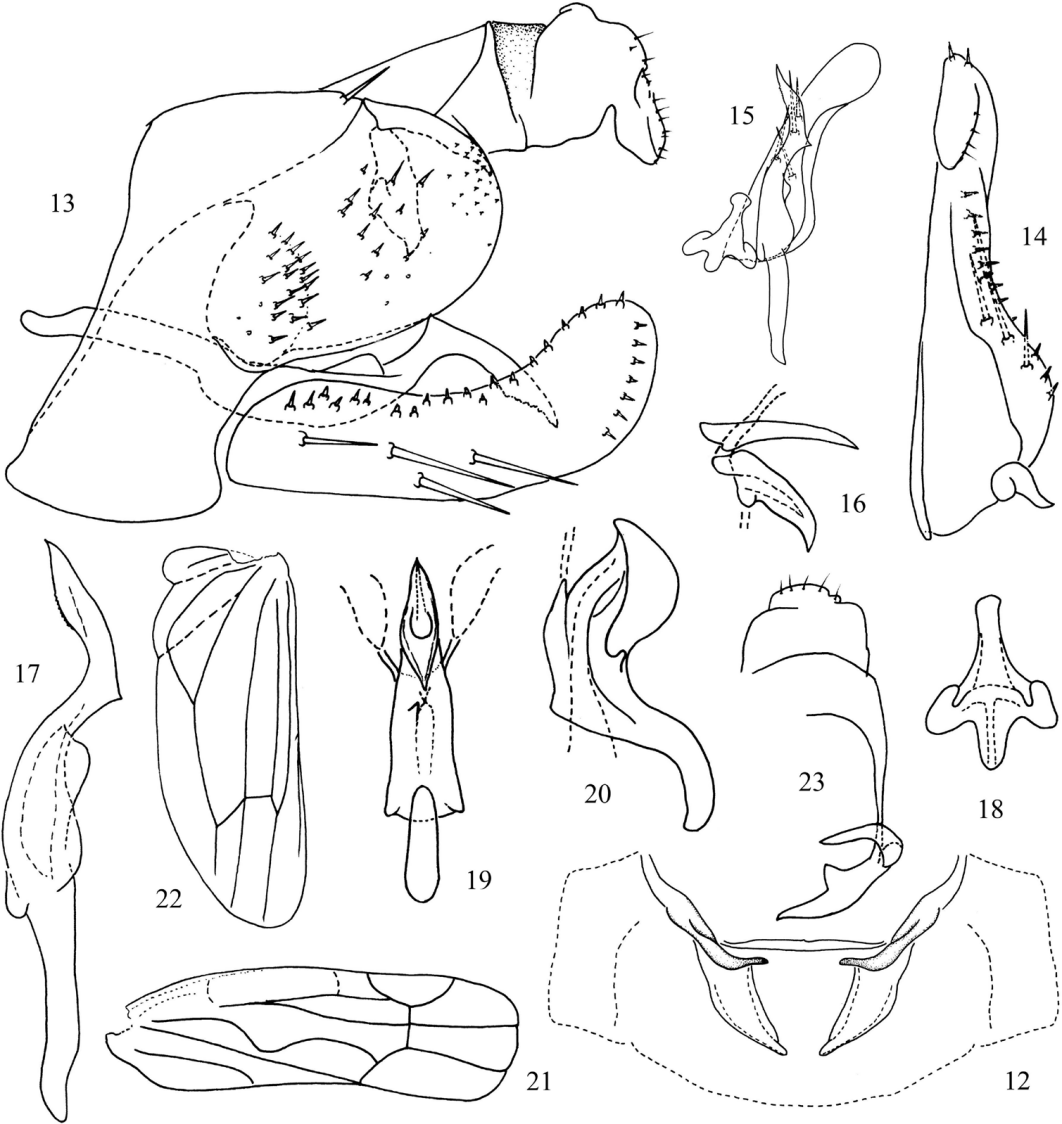
*Undulivenathaiensis* sp. n. **12** abdominal apodemes **13** genital capsule **14** subgenital plate **15** subgenital plate, style and connective **16** pygofer dorsal appendage (upper part) and an extension of anal tube appendage (lower part), lateral view **17** style **18** connective **19** aedeagus, ventral view (broken line indicates ligament attaching to pygofer dorsal appendage) **20** aedeagus, ventro-lateral view **21** venation of forewing **22** venation of hind wing **23** anal tube appendage with an apex extension and pygofer dorsal appendage, dorsal view.

Male abdominal apodemes short, not extending to hind margin of 3^rd^ sternite (Fig. 12). Male genitalia as in generic description with male pygofer dorsal appendage tapered distally; extension of anal tube appendage hook-like apically (Figs 16, 23). Pygofer lobe with one dorsal macrosetae (Fig. 13). Subgenital plate with group of 4 macrosetae laterally in basal half (Figs 13, 14). Style with 2^nd^ extension of apical process expanded at midlength thereafter tapered to acute apex (Fig. 17). Aedeagal shaft with single small subbasal tooth-like process on left side, shaft spindle-shaped in ventral view with sharp basal corners and pair of converging lateral flanges distally, dorsal surface distally keel-like (Fig. 19); dorsal apodeme small tapered to apex in lateral view and with two sharp distal corners connected with ligament to ventral pygofer process; preatrium moderately long (Figs 19, 20).

Hind margin of female 7^th^ sternite convex medially (Fig. 9).

**Measurements.** Body length, males 3.2–3.3 mm, females 2.7–2.8 mm.

#### Specimens examined.

Holotype: ♂, THAILAND, Kanchanaburi, Khuean Srinagarindra NP, Chong Kraborg, 14°29.972'N, 98°53.035'E 210m, Malaise trap, 4–11.ix.2008, coll. Boonnam & Phumarin (QSBG). Paratypes: 3♂♂, 3♀♀, same data as holotype; 4♂♂, THAILAND, Kanchanaburi, Khuean Srinagarindra NP, Tha Thung-na/Chong Kraborg, 14°29.972'N, 98°53.035'E 210m, Malaise trap 6–13.xi.2008, coll. Boonnam & Phumarin (INHS, SKS).

#### Remarks.

This species can be distinguished by external and male genitalia characters (see generic Remarks).

#### Etymology.

The species is named for the type locality, Thailand. The name is adjectival.

## Supplementary Material

XML Treatment for Undulivena

XML Treatment for Undulivena thaiensis
